# An unusual case of multiple hepatic and pulmonary abscesses caused by *Aggregatibacter aphrophilus* in a young man: a case report

**DOI:** 10.1186/s13256-020-02650-z

**Published:** 2021-02-04

**Authors:** Johannes Sumer, Sabine Haller, Mikael Sawatzki, Jan Kellner, Katia Boggian

**Affiliations:** 1 Division of Infectious Diseases and Hospital Epidemiology, Cantonal Hospital St. Gallen, Rorschacher Strasse 95, 9007 St. Gallen, Switzerland; 2 Division of Gastroenterology, Cantonal Hospital St. Gallen, Rorschacher Strasse 95, 9007 St. Gallen, Switzerland; 3 Division of Radiology, Cantonal Hospital St. Gallen, Rorschacher Strasse 95, 9007 St. Gallen, Switzerland

**Keywords:** *Aggregatibacter aphrophilus*, Liver abscess, Eubacterial PCR

## Abstract

**Background:**

*Aggregatibacter aphrophilus*, formerly known as *Haemophilus aphrophilus*, belongs to the HACEK organisms, a group of pathogens classically associated with infectious endocarditis. *A. aphrophilus* is a rarely found pathogen, though abscess formation in various organs has been described, typically due to spread from an infected heart valve. Here we describe the unusual case of multiple hepatic abscesses caused by *A. aphrophilus*.

**Case presentation:**

A 33-year-old Caucasian man presented at our hospital with fever and malaise, elevated inflammatory markers, and liver enzymes. Imaging was compatible with multiple liver and pulmonary abscesses, without evidence of endocarditis. Cultures of blood and liver abscess material remained without growth. Polymerase chain reaction finally revealed *Aggregatibacter aphrophilus* in the liver tissue. The patient recovered fully within 6 weeks of doxycycline treatment.

**Conclusions:**

There are only a few case descriptions of liver abscesses caused by *A. aphrophilus*. As a ubiquitous organism in the gastrointestinal tract, *A. aphrophilus* may reach the liver via the portal venous system, as well as through hematogenous spread from the oropharynx. HACEK organisms are notoriously difficult to grow on culture, which highlights the diagnostic importance of eubacterial PCR.

## Background

With this case report, we aim to demonstrate an unusual site of infection by *Aggregatibacter aphrophilus*, part of the HACEK group, in the form of multiple small liver abscesses, with the background of possible transmission via hematogenous spread from oropharynx or via the portal venous system and a potential infection through contact with animal saliva. Existing literature reports endocarditis as the most common manifestation of *A. aphrophilus* infection, with other sites of infection rarely described.

Furthermore, optimal antibiotic treatment and duration for hepatic abscesses with *A. aphrophilus* are not well established. In our case, the clinical response to antibiotic treatment with doxycycline for 6 weeks in total was excellent.

With our case report, we also aim to highlight the importance of diagnostic eubacterial polymerase chain reaction (PCR) in cultures without growth.

## Case presentation

A 33-year-old, previously healthy Swiss male was admitted to our hospital with 10-day history of fever and chills, accompanied by epigastric discomfort, nausea, and malaise. The patient had first presented to a regional hospital, where symptomatic treatment was initiated without lasting effect. Persisting fever and new-onset emesis led to presentation at our tertiary hospital. He worked as a haulier.

Past medical history was not significant, and he neither smoked nor drank alcohol on a regular basis. The patient had just returned from a week-long holiday in Austria, where among other activities he had visited a children’s zoo. His wife and 2-year-old daughter did not experience any illness.

On clinical examination, the patient presented with a temperature of 36.8 °C, blood pressure 90/59 mmHg, heart rate 100/minute, and oxygen saturation 98%. A small inguinal lymph node was noted. The rest of the clinical examination was unremarkable.

Laboratory examination at admission revealed a markedly elevated C-reactive protein (CRP) of 404 mg/l. The complete blood count showed a normal leukocyte count with neutrophilia of 86% and a left shift (17% band forms, increasing to 37% the following day). There was slight anemia (hemoglobin 130 g/l) and normal thrombocyte count (245 G/l). The transaminases [aspartate transaminase (AST) 95 U/l, alanine transaminase (ALT) 104 U/l] and alkaline phosphatase (156 U/l) were elevated 2–3-fold the upper limit of normal. Bilirubin (27 µmol/l) and international normalized ratio (INR) were slightly elevated with low albumin (28.5 g/l), indicating incipient impairment of liver synthesis function. Creatinine (87µmol/l) and pancreas amylase (33 U/l) were within the normal range. Antinuclear antibodies (ANA) were unremarkable. Leukocyturia (46/µl) and microhematuria (479/µl) were noted and interpreted as likely para-infectious changes in the absence of clinical evidence of urinary tract infection.

Treatment with doxycycline p.o. 100 mg bid was initiated on the assumption of an intracellular pathogen, and the fever disappeared within 3 days.

Aerobic and anaerobic blood cultures and serological tests for Epstein–Barr virus and cytomegalovirus taken at the regional hospital 5 days after onset of symptoms were negative. Further serology testing was negative for human immunodeficiency virus (HIV), hepatitis B, C, and A virus, cytomegalovirus, as well as influenza A and B virus. Due to the history of animal contact, serological tests for *Coxiella burnetii*, *Brucella*, *Leptospira interrogans*, *Francisella tularensis*, *Bartonella henselae*, and *Rickettsia rickettsii*/*conorii* and *typhi* were added, all of which came back negative.

**Fig. 1:**
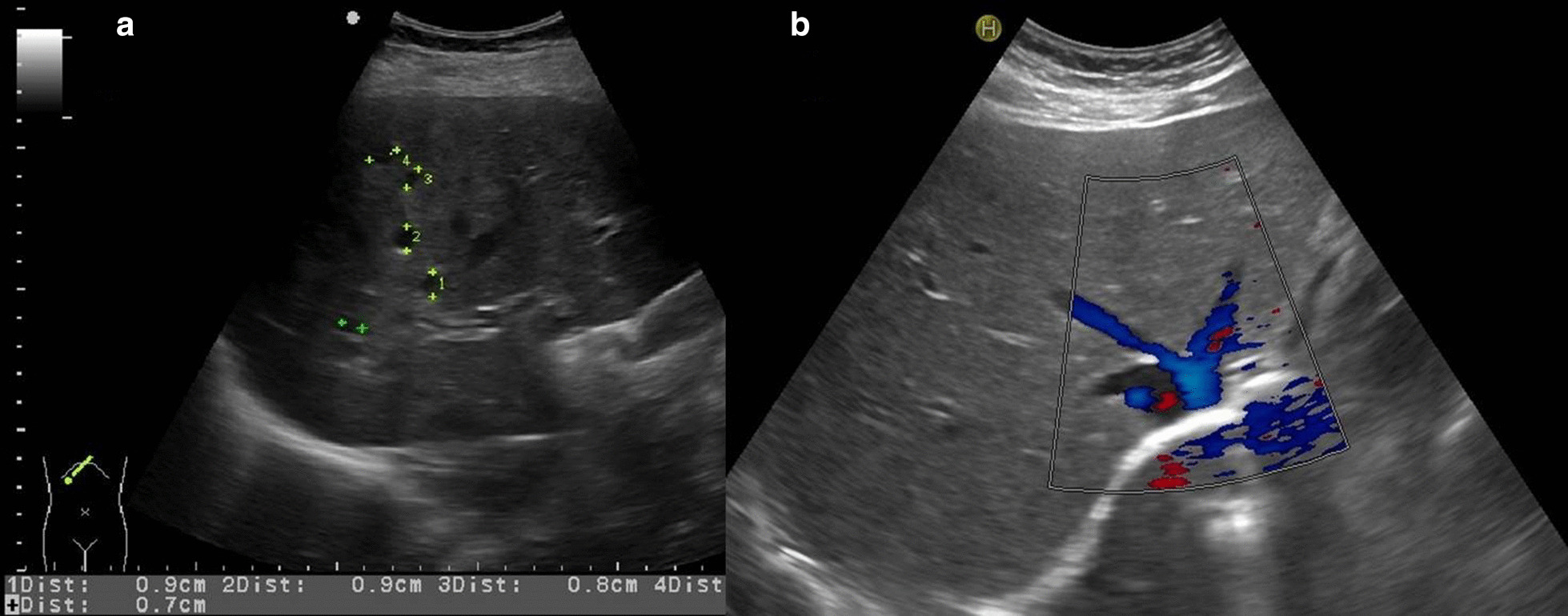
**a** B-mode ultrasound with hypoechoic liver lesions in the right liver lobe. **b** Normal liver tissue after successful antibiotic therapy

**Fig. 2:**
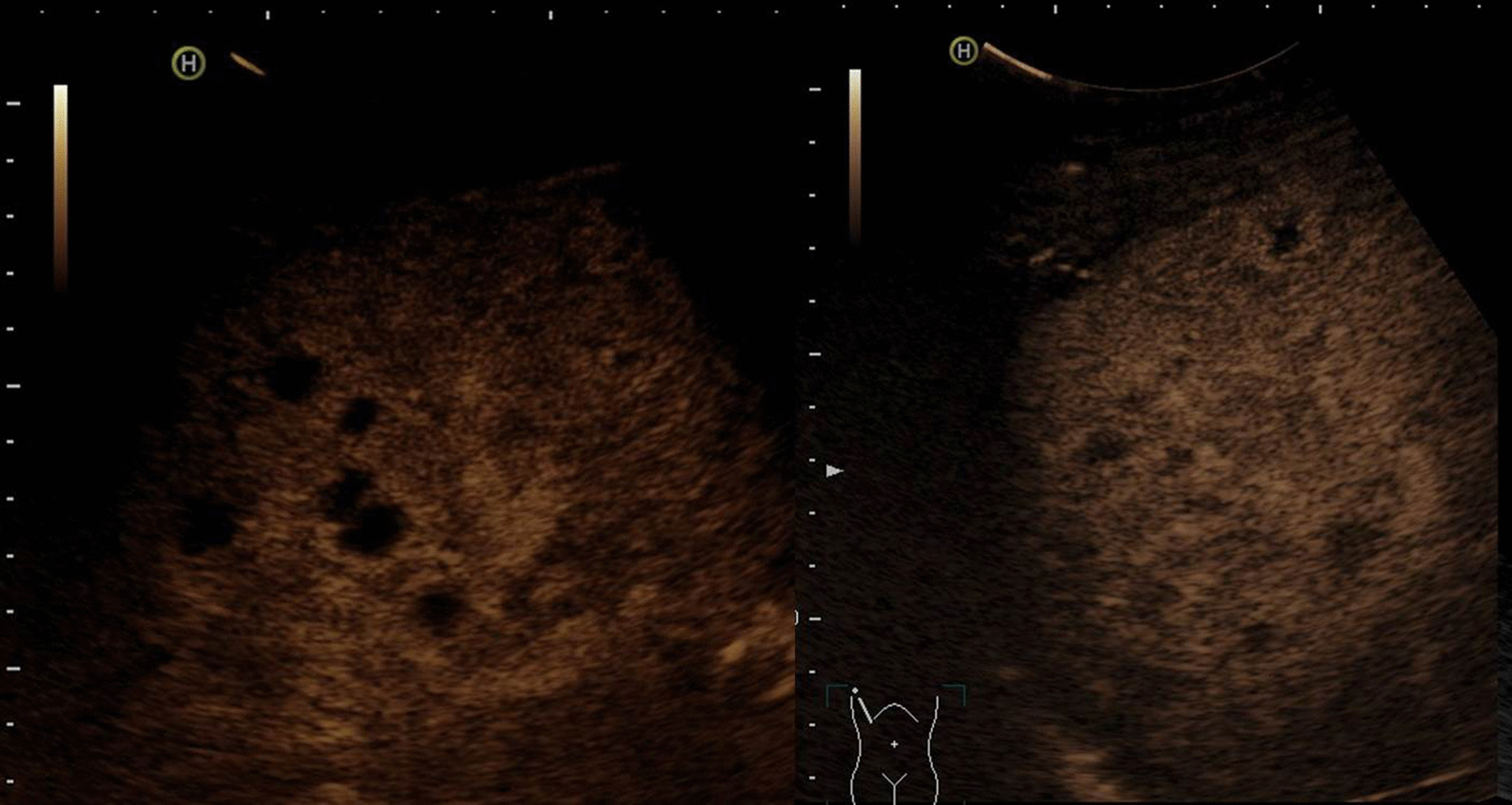
Contrast-enhanced ultrasound with suggested rim-like hyperechogenic contrast uptake due to hyperemia around the abscesses (stage IV). Due to missing arterial perfusion, there is no contrast uptake in the necrotic hypoechogenic lesions

A conventional abdominal ultrasound scan with contrast-enhanced ultrasound (CEUS) revealed disseminated small, hypoechoic liver lesions (Fig. 1a), partially with hyperechoic rim (Fig 2), measuring up to 1.8 cm in diameter. CEUS was performed for its superior sensitivity in the demarcation of liver abscesses, indicating stage IV pyogenic liver abscesses [1].

**Fig. 3:**
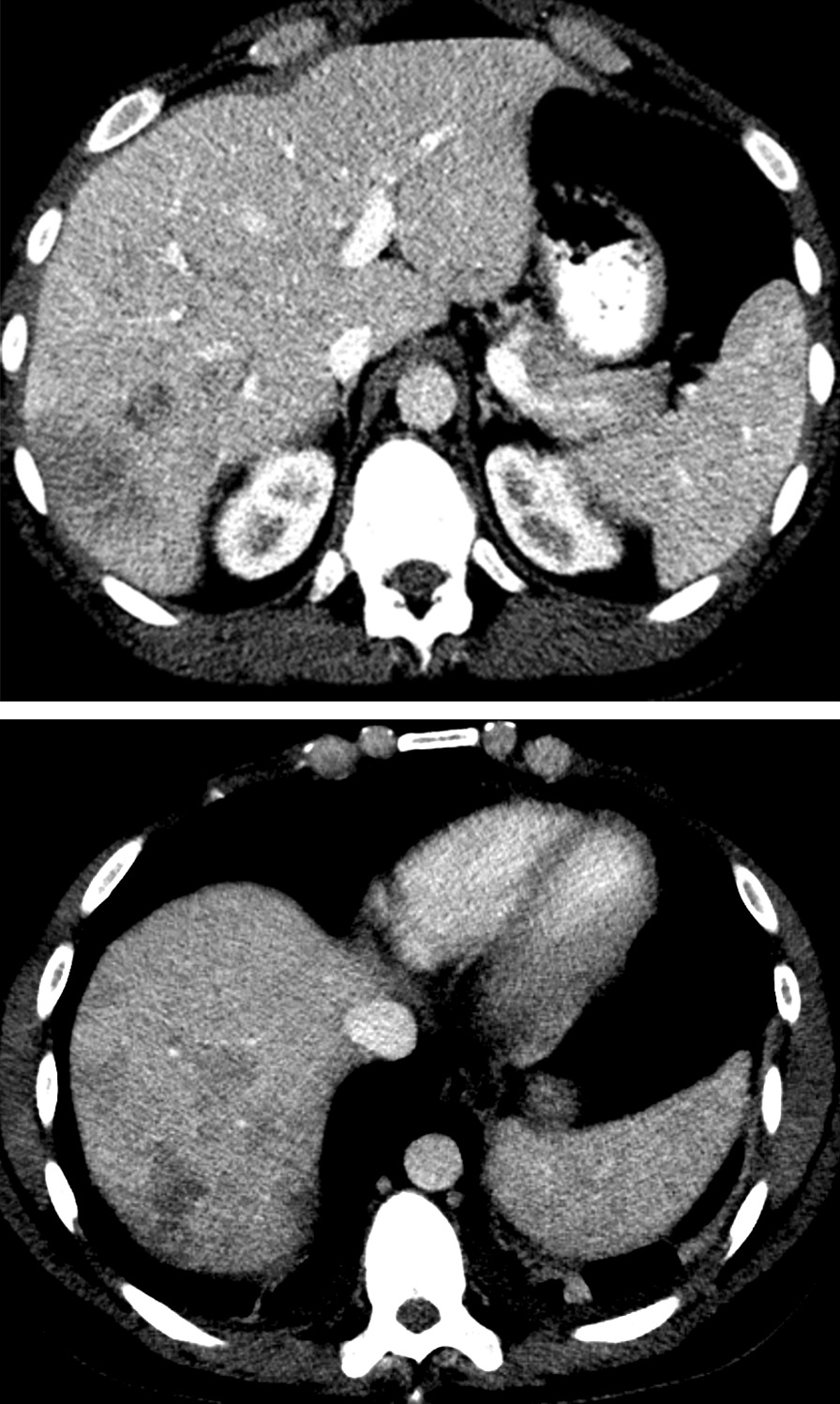
Computed tomography with oral and intravenous contrast agent, showing irregular hypodense liver lesions in segment VI and VII in portal-phase contrast-enhanced CT, corresponding to the ultrasound findings

A computed tomography (CT) scan confirmed multiple, partially confluent hypodense lesions of the liver consistent with the diagnosis of abscesses (Fig. 3). It also demonstrated bilateral small, nodular pulmonary lesions and slightly larger consolidations in the right apex (1.2 cm) and lingula (1.1 × 2.2 cm^2^), as well as hilar and mediastinal lymphadenopathy. Transthoracic echocardiography was normal.

Blood cultures taken on day 1 of admission remained without growth. On day 7, the patient underwent a percutaneous ultrasound-guided biopsy of one of the liver lesions, while under treatment with doxycycline. The histopathological examination revealed granulocytic inflammatory infiltrates and abscess formation. No microorganisms could be detected in Gram, periodic acid–Schiff (PAS), diastase-PAS, Grocott, and Ziehl–Neelsen stains.

Aerobic and anaerobic cultures of the liver abscess material obtained by biopsy remained without growth, including mycobacterial cultures. Accordingly, the Gram stain of the liver aspirate showed no microorganisms. Eubacterial PCR of the liver biopsy tissue with 16S ribosomal DNA (rDNA) sequencing finally revealed the presence of *Aggregatibacter aphrophilus*.

At an outpatient follow-up visit 1 month after hospital discharge, the patient was asymptomatic with normal inflammatory markers and liver function tests. The ultrasound scan found that the liver abscesses had completely resolved (Fig. 1b), with no residual splenomegaly. A repeat echocardiography showed no abnormalities, specifically no signs of endocarditis. Doxycycline was stopped after a total of 6 weeks of treatment.

## Discussion and conclusions

We report a case of a previously healthy young man with multiple hepatic and potentially pulmonary abscesses with *Aggregatibacter aphrophilus*. The patient responded well to antibiotic treatment with doxycycline given for 6 weeks. According to literature, *A. aphrophilus* is well known for endocarditis and there are only rare case reports about mostly solitary liver abscesses and other affected organs.

*Aggregatibacter aphrophilus*, formerly known as *Haemophilus aphrophilus* [2], belongs to the HACEK group and is a small, fastidious Gram-negative coccobacillus with colonies that are nonhemolytic, catalase-negative, and CO_2_-dependent and require X-factor (hemin) for growth. *Aggregatibacter aphrophilus* is part of the normal oropharyngeal commensal flora [3]. It has been documented as a rare cause of infections affecting various organs, although HACEK organisms typically cause endocarditis. In the case of our patient, there was no known predisposition for or evidence of endocarditis.

Despite the ubiquitous presence of *A. aphrophilus* in the gastrointestinal tract, invasive infection is infrequent, probably due to its low virulence. Hepatic abscess manifestation is rarely described according to our literature search. O'Bryan *et al.* describe a case of *H. paraphrophilus*, and a literature review identified a series of 10 cases with *Haemophilus* species causing infection of the liver and biliary system, in which most patients had predisposing conditions (e.g., hydatid cysts, previous cholecystectomy) [4]. In contrast, our patient did not have any comorbidities or predisposing factors. *A. aphrophilus* may reach the liver by ascending from the gastrointestinal tract, via the portal venous system, or by hematogenous seeding following oropharyngeal colonization [4]. In our patient, the most probable explanation may be daily dental hygiene with subsequent bacteremia leading to pulmonary and hepatic abscess formation. In three case reports [5–7], close contact to a dog was reported, which may account for a possible source [8]. In one case, *Aggregatibacter* was found in the saliva of the dog, making it a likely vector [6]. Due to the patient’s history of visiting a children’s zoo, contact with animal saliva—potentially contaminated with *Aggregatibacter*—should be considered as a possible infection route.

An interesting and unusual feature of our case is the fact that the infection presented in the form of multiple small abscesses within the liver. Previous case reports described solitary abscess formation [9–11], while in one case two large abscesses formed [10]. Such large liver abscesses are presumably due to portocaval spread. In our patient, multiple tiny pulmonary lesions were additionally described on CT scan, most likely representing pulmonary abscesses. Hence, we presume hematogenic spread of *Aggregatibacter*, leading to the formation of pulmonary and hepatic abscesses. Regarding the pulmonary abscesses, unfortunately we do not have any sample of the respiratory tract to provide microbiological or histological evidence.

Other rare manifestations of *Aggregatibacter aphrophilus* have been described as brain abscesses [12], vertebral osteomyelitis, spinal epidural abscess [13], mediastinitis [14], pulmonary abscess [14, 15], meningitis [15], endophthalmitis [16], liver [9], spleen [17], retroperitoneal, psoas and scrotal abscesses [18], as well as recurrent empyema [19].

Bacteria of the HACEK group are known to be fastidious organisms that show a characteristic slow growth. Standard microbiological cultures most often remain negative, adding to the complexity of identifying this rarely encountered organism. This highlights the diagnostic importance of eubacterial PCR in cases when standard medium cultures remain inconclusive and infections with difficult-to-culture bacteria may be missed without DNA sequencing.

Optimal antibiotic treatment and duration for hepatic abscesses are not well established. Resistance to ampicillin with beta-lactamase production has infrequently been reported for *Aggregatibacter* and *Haemophilus* [20, 21]. We continued the empirical treatment of our patient with doxycycline past his discharge since he tolerated this antibiotic well and showed a good clinical response. The duration of therapy for *Aggregatibacter* causing hepatic abscesses is not well established due to a limited number of reported cases. In general, treatment for pyogenic liver abscesses is specified with variable duration of 4–6 weeks according to present literature [22–24]. Presuming hematogenic spread, we treated our patient for 6 weeks, as recommended for HACEK endocarditis.

Short courses (2 weeks) of therapy after percutaneous drainage of hepatic abscesses have been successful in a small series of patients, but most series have reported recurrence of abscess even after more prolonged courses [25]. In our case, repeat transthoracic echocardiography as well as abdominal ultrasound at the end of treatment showed no abnormalities. In conclusion, *A. aphrophilus* can cause abscesses in different locations and should be considered when microbiological samples remain negative. When cultures remain without growth, 16S rDNA PCR performed directly on abscess fluid can be useful.

## Data Availability

All patient data that support this case report are included in anonymized form in the published article.
